# Supplementary material for: Mechanical response design of arch seat foundation under complex traffic loading
Source: PLoS One. 2025 Dec 22;20(12):e0338429. doi: 10.1371/journal.pone.0338429 (PMC12721545; doi:10.1371/journal.pone.0338429)
Supplement: Attachment — Submitted filename: Response to reviewers.docx [file pone.0338429.s002.docx]

**Replies to the reviewers’ comments (PONE-D-25-51129)**

**Reviewer #1:**

The paper addresses a practically important topic: the mechanical response of arch-seat foundations of extra-large arch bridges subjected to realistic combinations of traffic, temperature, wind and braking loads. The authors construct a large-scale 3-D finite-element model and examine stresses, axial forces, bending moments, and soil plasticity. The study is timely, but the current manuscript still needs significant improvement in clarity,

completeness of methodology, and discussion of novelty.

1. Significance and Novelty

• The paper is relevant to long-span bridge foundation engineering.

• The use of a full-scale 3-D model with coupled load combinations is worthwhile.

• However, the novelty is not convincingly highlighted. Similar FEM-based investigations of arch-seat or abutment foundations under multi-field loading already exist.

• The manuscript should explain more clearly what is new—for example: the systematic comparison of vertical vs. slanted piles under realistic combined loads, the explicit inclusion of temperature gradients and braking forces, and the link between soil plasticity concentration and foundation design recommendations.
**Reply:**

Thanks for your recognition and suggestions. The authors carefully reviewed the shortcomings of previous studies and described the innovation of this study in the revised manuscript based on the reviewer's suggestions (pages 2-5, lines 36-89): *Recent years have seen considerable research on arch seat foundations. In the construction domain, Qian et al. [5] introduced a stepped excavation method combined with smooth blasting to address waste removal difficulties in steep terrain. Li et al. [6], Tian et al. [7], and Li et al. [8] investigated three complex geological settings-water-rich soft rock, jointed rock slopes, and karst foundations-revealing deformation mechanisms of arch seat foundations, risks of plastic zone penetration in slopes, and the stress amplification effect in karst areas. Their proposed countermeasures, including dewatering infiltration control, bolt-grouting reinforcement, and karst concrete backfilling, helped address technical challenges in controlling foundation deformation under complex ground conditions. However, these studies mainly focused on construction strategies for specific geologies and did not compare the mechanical performance of different pile types under combined loading. In studies concerning long-term stability, Yang et al. [9] used creep tests and numerical simulations to quantify stress concentration and deformation characteristics at the contact interface of a solution-collapse gravel rock foundation, establishing evaluation criteria for long-term safety of arch seat foundations in soft rock areas. For local stress and seismic performance, Zhao [10] applied a global-local finite element approach to analyze stress control during construction but did not include temperature gradients-particularly the coupling of vertical and horizontal gradients-or train braking forces in the load combinations, leading to deviations from real service conditions. Gorini et al. [11, 12] developed an efficient dynamic simulation method for arch seats, overcoming the low computational efficiency of conventional continuum models. Al-Homoud and Whitman[13] experimentally and numerically validated a seismic response model for rigid arch seats governed by tilting, providing key data for seismic design. Balla and Manandhar [14] compared traditional and finite element methods (FEM), demonstrating FEM's economic and accuracy benefits in computing foundation internal forces. However, their study focused solely on internal forces and did not correlate plastic strain localization in the soil-rock improved zone with the optimization of foundation design parameters.*

*Although existing research has improved understanding of construction techniques in complex geology, long-term stability in soft rock, and mechanical behavior under specific loads, several limitations persist, hindering the translation of research outcomes into engineering practice. First, the combined influence of complex traffic loads-such as temperature gradients (especially the coupling of longitudinal and transverse gradients)[15, 16], braking forces[17-19], and wind loads[20, 21] -has not been adequately considered. Most studies analyze individual loads in isolation or fail to integrate them into a unified coupled framework. As a result, the load-transfer mechanism in the arch seat foundation–soil–structure system deviates significantly from actual bridge behavior, making it difficult to accurately capture interactive responses under superimposed loads. Moreover, key mechanical behaviors under coupled loads remain poorly understood. In particular, systematic analysis is lacking on stress concentration patterns across different foundation components and on the internal force distribution differences between vertical and slanted piles under the same loading. This gap leaves pile type selection and anti-deformation design without a solid scientific basis. Furthermore, few studies have adopted real engineering projects as a basis for establishing full-scale models that integrate the main beam, arch footings, arch seat foundation, and slope. The relationship between plastic concentration in the remodeled soil-rock zone and foundation design parameters is also rarely examined. Consequently, many research findings fail to offer systematic guidance for precise engineering design and long-term operation and maintenance.*

*Given the above issue, this study develops a full-scale three-dimensional finite element model (3D FEM) based on a large reinforced concrete arch bridge project in China, integrating the main beam, arch feet, arch seat foundation, and surrounding slope. The model comprehensively incorporates complex traffic load combinations, including dead and live loads, train-induced vibrations, braking forces, global thermal effects, coupled longitudinal and transverse temperature gradients, and wind loads, as well as the embedment condition of the arch seat within the slope. A systematic analysis is conducted on the mechanical responses of the arch seat cap, vertical and slanted piles, and surrounding soil-rock mass. The study clarifies the stress distribution, deformation characteristics, load transfer mechanisms, and load sensitivity of different components under complex traffic conditions. Based on the results, targeted design optimizations and long-term maintenance strategies are proposed, with the aim of providing scientific support for the design and preservation of bridge engineering structures.*

2. Numerical Modeling

Strengths

• Clear description of geometry, boundary conditions, and load cases (S1–S3).

• Sensible mesh-sensitivity study to select an efficient mesh.

**Reply:**

Thank you very much for your recognition.

Issues & Suggestions

1) Constitutive model:

(a) The paper uses the extended Drucker–Prager model but does not give the hardening law in full detail (stress–strain curve, yield evolution).

**Reply:**

Thanks for your comments and suggestions. The authors provide a schematic diagram of the yield surface (Fig. 5) and plastic potential surface (Fig. 6) of the DP model on pages 12-13 of the revised manuscript. Also, the hardening law was added to the revised manuscript according to the reviewer's suggestion (pages 11-13, lines163-193): *In the numerical analysis presented, the extended linear Drucker-Prager (DP) model is employed to describe the stress-strain relationship of the geotechnical material of the slope surrounding the arch seat foundation. To minimize boundary effects, the geometric model is set to a relatively large size, so an elastic constitutive model (0 m-130 m) is used for soil layers at greater distances without affecting the calculation results. The yield surface F of the DP model (Fig. 5) is defined by the following equation*

$F=t-ptan\beta-d=0$ *(1)*

*To reflect the influence of the intermediate principal stress, the yield surface in the DP model is defined by a parameter t, representing the deviatoric stress, and is given by*

$t=\frac{q}{2}\left[1+\frac{1}{k}-\left(1-\frac{1}{k}\right)\left(\frac{r}{q}\right)^{3}\right]$ *(2)*

*where β is the inclination of the yield surface in the p-t stress space, related to the friction angle ψ, k is the ratio of the triaxial tensile strength to triaxial compressive strength, reflecting the influence of the intermediate principal stress on yielding, and d is the intercept of the yield surface on the t-axis in the p-t stress space, given by*

$d=\left(1-\frac{1}{3\tan\beta}\right)\sigma_{c}$ *(3)*

***Fig. 5*** *Yield surface of the linear DP model[25]*

*where σ_c_ is the uniaxial compressive strength. The DP model uses a non-associated flow rule, with the plastic potential surface G (Fig. 6) given by*

$G=t-ptan\Psi$ *(4)*

*where Ψ represents the dilatancy angle. The extended DP model in ABAQUS allows for the expansion (hardening) or contraction (softening) of the yield surface, with hardening being applied in the numerical analysis presented. The variation in the yield surface size is controlled by the equivalent stress* $\overline{\sigma}$*, which is determined by the relationship between equivalent stress and equivalent plastic strain* $\overline{\varepsilon}^{pl}$*. The equivalent plastic strain is given by*

$\overline{\varepsilon}^{pl}=\int\Delta\overline{\varepsilon}^{pl}dt$ *(5)*

*Fig. 6 Plastic potential surface of the linear DP model[25]*

*For the linear DP model, when compressive hardening behavior is applied, the increment of equivalent plastic strain in ABAQUS is calculated by*

$d\overline{\varepsilon}^{pl}=\left|d\varepsilon_{11}^{pl}\right|$ *(6)*

(b) Justify the choice of friction angle 30°, dilatancy 15°, and whether strain-softening was considered for mudstone.

**Reply:**

Thanks for your comments and suggestions. In the revised manuscript, the authors provide the axial stress-strain curves of the field mudstone samples according to the reviewer's suggestion in 1 (a) (including the experimental and numerical results under unconfined pressure and the numerical results under low confining pressure). The results show that the numerical results are in good agreement with the experimental results, indicating that the selection of geotechnical parameters is reasonable (in fact, the friction angle and dilatancy angle are provided in the project's investigation report). Of course, the strain-softening characteristics of mudstone are taken into account in the numerical simulation, which can be proved by the axial stress-strain curve in the Fig. 7. Details are as follows (pages 13-14, lines 198-210): *Figure 7 shows the comparison between the experimental results and numerical results of the uniaxial compression test of mudstone. Under unconfined pressure conditions, the axial stress of mudstone increased linearly with axial strain. When the stress reached the peak strength (i.e., uniaxial compressive strength), microcracks inside the rock initiated and propagated, and the stress decreased significantly as strain continues to increase, exhibiting obvious strain softening behavior. This phenomenon was also well captured in the numerical simulation under low confining pressure. The axial stress-strain curve of mudstone obtained from the experiment shows a good fitting effect with the numerical results, indicating that the constitutive model and geotechnical parameters selected in the numerical simulation of this paper are reasonable and applicable.*

Fig. 7 Axial stress-strain curves of field mudstone samples: comparison between experiments and numerical simulations

2) Load application: Forces and moments at reference points are tabulated, but the derivation from code-based design loads is not shown; a brief explanation or a supplementary note would improve transparency.

**Reply:**

Thanks for your comments and suggestions. The authors fully appreciate the reviewer's intention to enhance the integrity of load application, which was also a main consideration during the manuscript preparation. However, the current version already represents the maximum extent of what we can achieve. The specific reasons are as follows:

This bridge with a total length of nearly 1000 meters, is of considerable scale, involving structural components of various geometric types and materials with different properties. Meanwhile, the structural analysis of the bridge includes numerous complex loads, which correspond to multiple design codes and standards. Therefore, the structural analysis of this bridge was not completed by one or several individuals; instead, independent calculations were conducted by teams from multiple specialized companies. The stress state of each node of the entire bridge under different load combinations was finally determined after comparative verification. In summary, the derivation of design loads is a large-scale undertaking, far beyond the scope and content limitations of this paper.

The extra-long-span bridge involved in this study has not yet been fully completed and is subject to a high level of confidentiality. The authors must comply with the confidentiality agreements and ethical guidelines formulated by the competent authorities. We do not have access to the load design documents, let alone publicly publish them-this also prevents us from providing numerous materials, including bridge design drawings and geological survey reports. Even Tables 4 to 6 represent information obtained through the authors' best efforts.

Based on the two main reasons outlined above, we hope the reviewer can understand the authors' inability to provide further supplements.

3) Boundary conditions: The constraints at the far field of the slope need justification to ensure minimal reflection and realistic stress transmission.

**Reply:**

Thanks for your comments and suggestions. The authors tried other constraints, but the gravity field could not be applied smoothly. Therefore, we chose the constraints of this paper. For the boundary effect, the authors added the relevant description on page 9, lines 138-144 of the revised manuscript according to reviewer’s suggestions: *The minimum distance from vertical piles to the X-direction boundary is approximately 230 m (>31D_v_, D_v_ represents the diameter of the vertical pile), and the minimum distance to the Z-direction boundary is approximately 90 m (>12D_v_); the minimum distance from inclined piles to the X-direction boundary is approximately 200 m (>24D_s,a_, D_s,a_ represents the average width of the inclined pile), and the minimum distance to the Z-direction boundary is approximately 82 m (>10D_s,a_). Under such geometric conditions, the influence caused by boundary effects is significantly reduced, ensuring minimal reflections and realistic stress transmission[22, 23].*

Reference:

Yang J. Influence zone for end bearing of piles in sand. J Geotech Geoenviron. **2006**, 132(9), 1229-1237. <https://doi.org/10.1061/(asce)1090-0241(2006)132:9(1229)>.

Yu F, Yang J. Base Capacity of Open-Ended Steel Pipe Piles in Sand. J Geotech Geoenviron. **2012**, 138(9), 1116-1128. <https://doi.org/10.1061/(asce)gt.1943-5606.0000667>.

4) Verification: The comparison between numerical and theoretical axial-force distribution is useful; more such verification (e.g., deflection of arch foot) would increase confidence.

**Reply:**

Thanks for your comments and suggestions. According to the reviewer's suggestions, the comparison of the stress-strain curves of the field mudstone samples is shown in Fig. 7 to verify that the constitutive model and parameter selection are reasonable and applicable (pages 13-14, lines 198-206): *Figure 7 shows the comparison between the experimental results and numerical results of the uniaxial compression test of mudstone. Under unconfined pressure conditions, the axial stress of mudstone increased linearly with axial strain. When the stress reached the peak strength (i.e., uniaxial compressive strength), microcracks inside the rock initiated and propagated, and the stress decreased significantly as strain continues to increase, exhibiting obvious strain softening behavior. This phenomenon was also well captured in the numerical simulation under low confining pressure. The axial stress-strain curve of mudstone obtained from the experiment shows a good fitting effect with the numerical results, indicating that the constitutive model and geotechnical parameters selected in the numerical simulation of this paper are reasonable and applicable.*

Fig. 7 Axial stress-strain curves of field mudstone samples: comparison between experiments and numerical simulations

In addition, the comparison of the bending moment distribution of vertical pile B is added to the revised manuscript to further enhance confidence (pages 16-17, lines 227-246): *As illustrated in Fig. 9, the coarse mesh (Case 1) did not simulate the mechanical response of the vertical pile accurately, especially near the head of the pile cap, where the deviation from the theoretical results exceeded 1.4 MN. The numerical results of Case 2 differed from those of Case 1, indicating that the results were still affected by the mesh size under this condition. The results of Case 3 showed good consistency with the theoretical calculations, validating the feasibility of the numerical model. Although there was some deviation in capturing load transfer at 20% and 80%, the error remained within 4%. In Case 4, the mesh was further refined, but no significant change in results was observed compared to Case 3, indicating that the computational results were insensitive to mesh size under these conditions. Additionally, in terms of computational cost (Table 2), Case 4 showed an approximately 45% increase in cost compared to Case 3, while the accuracy gain was minimal. Figure 7 presents the bending moment distribution of vertical pile B under different mesh sizes. Similar to the axial force distribution, the bending moment distribution under Case 1 exhibited a significant deviation from the theoretical values, concentrated at the junction of the vertical pile and the arch abutment cap as well as the lower part of the pile. The results of Cases 2 to 4 achieved good agreement with the theoretical values in the lower part of the pile, while deviations still exist in the upper part of the pile. However, as the mesh density increases, this deviation gradually decreases. Through a comparative observation of Figs 9 and 10, it can be found that the mesh size of Case 3 achieves the optimal balance between "simulation accuracy" and "computational efficiency". Therefore, this mesh size is adopted for all subsequent computational analyses.*

**Fig. 9** Axial force distribution of vertical pile A corresponding to different mesh sizes

**Fig. 10** Bending moment distribution of vertical pile B corresponding to different mesh sizes

5) Reproducibility: I strongly recommend that the authors provide the Abaqus input file (*.inp) or the CAE file as supplementary material. This would allow reviewers and future readers to reproduce the results and examine modeling details such as contact definitions, element types, and load steps.
**Reply:**

Thanks for your comments and suggestions. The authors fully appreciate the reviewer's perspective on promoting scientific progress through open access to knowledge. Following the reviewer's suggestion, we have provided the preliminary input file (*.inp) for Case S1 as an attachment within the framework of the competent authority's regulations and ethical guidelines, to facilitate reviewers and future readers to reproduce the results and examine modeling details.

3. Results and Discussion

1) The contour plots of stresses and strains are clear, but the captions should define symbols (e.g., VPA, SPA) and load cases for self-containment.
**Reply:**

Thanks for your comments and suggestions. Figs. 11-13, 16, and 17 in the revised manuscript have been modified based on the reviewers' suggestions, and some explanations have been added to enhance the readability of the figures. The details are as follows:

Fig. 11 (page 21, line 307):

**Fig. 11** Maximum principal stress contour plot for the arch seat foundation

Fig. 12 (page 23, line 331):

**Fig. 12** Maximum principal stress contour plot for the slanted piles

Fig. 13 (page 25, line 354):

**Fig. 13** Maximum principal stress contour plot for the vertical piles

Fig. 16 (page 31, line 459):

**Fig. 16** Equivalent plastic strain in the soil and rock mass surrounding the arch seat foundation.

Fig. 17 (page 32, line 461):

**Fig. 17** Vertical settlement of the soil and rock mass surrounding the arch seat foundation

2) The physical interpretation is reasonable, but in some places it is descriptive rather than analytical. For example:

(a) Explain why tensile stresses concentrate at the cap-arch-foot junction (geometry discontinuity, stiffness contrast).

**Reply:**

Thanks for your comments and suggestions. The more detailed analysis has been added to pages 19-20, lines 274-285 of the revised manuscript based on the reviewers’ suggestions and guidance: *As shown in Fig. 11, under the three complex traffic load conditions (S1-S3), a high tensile stress concentration zone appears on the inner side of the connection between the arch foot and the pile cap, with relatively smooth compressive stress on the outer side. The load transfer path is concentrated at the arch foot-pile cap connection interface. This interface serves as the "force flow hub" between the arch ring and the arch abutment foundation. Due to the significant sudden changes in stiffness and geometric structure between the arch ring box chamber and the arch seat cap, the axial force, bending moment, and shear force transmitted by the arch ring undergo load redistribution at this interface. Such stiffness discontinuity directly leads to severe concentration of stress flow inside the interface. Furthermore, the connection between the arch springing and the cap results in material deformation incompatibility (differences in elastic modulus and Poisson's ratio), and the curved surface of the arch springing generates radial tensile stress components. Both of these factors collectively induce the superposition of tensile stresses inside the interface.*

(b) Discuss potential cracking or fatigue implications of repeated traffic loading.

3) The engineering recommendations (steel reinforcement, grouting, monitoring) are sensible but would benefit from a clearer link to the numerical findings.

**Reply:**

Thanks for your comments and suggestions. In response to the two issues mentioned above, the authors added a more detailed analysis to the revised manuscript based on the reviewers' suggestions (pages 20-25, lines 285-354): *Under condition S2, the peak tensile stress reaches its highest value (23.70 MPa). This results from the combined effect of the additional bending moment induced by the transverse temperature gradient and the horizontal wind load during vehicle presence, leading to a steep stress gradient. In condition S1, the peak tensile stress is 21.38 MPa, accompanied by a relatively minor additional bending moment due to vertical temperature gradients, along with longitudinal braking forces. These contribute to a narrower stress concentration zone. Under condition S3, the peak tensile stress is 21.89 MPa. In the absence of vehicle loads, the stress distribution is more uniform. Among the three load combinations, the maximum compressive stress occurs under condition S3 (-7.99 MPa). The presence of compressive stress on the outer side of the foundation suggests that wind load exerts a more pronounced lateral compression effect when no vehicle load is applied. Under this scenario, the vertical compressive stress contribution from the primary load decreases, thereby increasing the proportion of horizontal compressive stress. The compressive stress values decrease sequentially in conditions S2 (-7.25 MPa) and S1 (-0.71 MPa). In condition S1, braking forces acting longitudinally contribute minimally to horizontal compressive stress at the foundation. In addition, vertical temperature gradients enhance the tensile tendency of the foundation, partially offsetting the compressive stress and resulting in a lower absolute value of compressive stress. Such cyclic and repeated action of high tensile stress tends to induce the initiation and fatigue propagation of microcracks under repeated traffic loads. In the long term, this may reduce the effective force transfer performance of the interface, posing a potential threat to the overall durability of the arch bridge. To mitigate this, engineering measures such as optimizing the reinforcement detailing at this interface (e.g., increasing bar diameter and density based on the stress concentration magnitude observed in Fig. 11) and deploying long-term strain monitoring systems to track stress evolution are crucial.*

**Fig. 11** Maximum principal stress contour plot for the arch seat foundation

*As shown in Fig. 12, under the three traffic load conditions (S1-S3), tensile stress is concentrated at the connection between the slanted piles and the foundation, while compressive stress localizes at the pile toes; no scattered high-stress zones are observed. In condition S1, the maximum tensile stress at the left slanted pile SPA reaches 0.93 MPa. This is attributed to longitudinal deformation discrepancy between the pile cap and the slanted piles induced by the vertical temperature gradient, combined with the longitudinal braking force and primary load, which together lead to a significant concentration of longitudinal force at SPA and a pronounced stress gradient. Under condition S2, the maximum tensile stress at SPA is 0.89 MPa, with a broader stress concentration zone resulting from transverse loading, though the peak value is slightly lower than in S1. In condition S3, the right slanted pile SPB exhibits a maximum tensile stress of 0.79 MPa. Absence of vehicle load reduces the load combination effect, resulting in a wider tensile stress concentration zone and a more gradual stress gradient. The slanted piles are constructed of C40 concrete, which has a characteristic axial tensile strength of approximately 2.39 MPa. In all cases, the peak tensile stress remains well below this limit, indicating that the pile body operates within the elastic range. Additionally, the embedded reinforcement helps optimize stress distribution and improves structural ductility. However, the cyclic stress action induced by repeated traffic loads may still induce fatigue damage. Especially in stress concentration zones, the steel-concrete interfaces are prone to the accumulation of fatigue cracks. Although the current stress level has not reached the ultimate strength, the long-term fatigue effects should be incorporated into the structural full-life assessment. Based on the stress distribution (e.g., the region of rapid stress gradient change in SPA), refining the reinforcement layout at the slanted pile-foundation connection (e.g., adopting lap splices or additional stirrups) and implementing periodic crack width monitoring, which directly responds to the fatigue risk indicated by the stress cycling characteristics.*

**Fig. 12** Maximum principal stress contour plot for the slanted piles

*Figure 13 presents the maximum principal stress contours of the vertical piles. Under all three conditions, the principal stress distribution exhibits a pronounced concentration at the pile head, with the stress gradient diminishing along the pile shaft. In condition S1, the maximum principal stress at the left vertical pile (VPA) reaches 2.74 MPa. The pile head region, subject to constraint from longitudinal deformation induced by the vertical temperature gradient, forms the core stress concentration zone. Under condition S2, the maximum principal stress is approximately 2.57 MPa. The inclusion of transverse temperature gradients and vehicular wind load causes the stress concentration zone to extend toward the upper-middle section of the pile. Although the peak stress is slightly lower than in S1, the coupled loading effect leads to more pronounced disruption in the lateral stress distribution along the pile. In condition S3, the maximum principal stress decreases to 2.26 MPa. In the absence of vehicle load, the stress concentration zone becomes more diffuse, though the pile head remains a stress-critical region. Under repeated traffic loads, cyclic stress concentration at the pile head, as quantified by the peak values (up to 2.74 MPa in S1), tends to induce fatigue cracking. Gradual propagation of microcracks weakens the effective cross-sectional stiffness of the pile shaft, while the stress redistribution of steel reinforcement and crack inhibition effect are particularly critical in delaying the fatigue damage process. Specifically, engineering practices should focus on enhancing the pile head reinforcement (e.g., increasing longitudinal bar diameter and adding confinement reinforcement) and deploying stress-strain gauges at the pile head, as the numerical findings (stress gradient and concentration magnitude) directly justify these measures for long-term fatigue resistance. This also highlights the necessity of long-term monitoring and fatigue performance optimization for the pile head region.*

**Fig. 13** Maximum principal stress contour plot for the vertical piles

4. Writing and Presentation

1) The manuscript is generally readable but would benefit from language editing to reduce repetition and improve grammar.

**Reply:**

Thanks for your comments and suggestions. The authors revised the entire manuscript to improve the paper's language.

2) The abstract is somewhat long; consider shortening while keeping motivation, approach, main findings, and practical implications.

**Reply:**

Thanks for your comments and suggestions. Revisions have been made on pages 1-2, lines 12-24 of the revised manuscript according to the reviewer's comments: *The mechanical response of the arch seat foundation of extra-long-span arch bridges under complex traffic loads has not been fully understood. To address this gap, this study develops a field-scale three-dimensional finite element model (3D FEM) of the arch seat foundation based on a large- span arch bridge project in China. Accounting for complex traffic loads-including longitudinal and transverse temperature gradients, braking forces, and wind loads-a detailed analysis was performed on the stress distribution of the foundation, load transfer of vertical and slanted piles, and stress-strain behaviors of the surrounding rock. The results show that the maximum tensile stress in the foundation is localized in the connection zone between the arch abutment cap and the arch springing. Tensile forces are most pronounced at the top of vertical piles, while the maximum bending moment occurs at the pile heads. Furthermore, plastic strain and settlement of the soil-rock mass are concentrated in the interconnected regions among the arch springing, cap, and main beam. Thus, it is critical to optimize reinforcement design at these key locations, implement long-term stress and strain monitoring, and mitigate the initiation and fatigue propagation of microcracks.*

3) Figures 7–13 are informative; consider including the scale bars and marking critical locations (pile head, pile tip, arch foot).

**Reply:**

Thanks for your comments and suggestions. Figs. 11-13, 16, and 17 in the revised manuscript have been modified based on the reviewers' suggestions, and some explanations have been added to enhance the readability of the figures. The details are as follows:

Fig. 11 (page 21, line 307):

**Fig. 11** Maximum principal stress contour plot for the arch seat foundation

Fig. 12 (page 23, line 331):

**Fig. 12** Maximum principal stress contour plot for the slanted piles

Fig. 13 (page 25, line 354):

**Fig. 13** Maximum principal stress contour plot for the vertical piles

Fig. 16 (page 31, line 459):

**Fig. 16** Equivalent plastic strain in the soil and rock mass surrounding the arch seat foundation.

Fig. 17 (page 32, line 461):

**Fig. 17** Vertical settlement of the soil and rock mass surrounding the arch seat foundation

4) Check that all references are complete and that the most recent related work is cited.

**Reply:**

Thanks for your comments and suggestions. Based on the reviewers' suggestions, the authors checked the completeness of the references. At the same time, while ensuring correct citation, we tried to consider the latest research. The updated references are as follows (pages 34-37, lines 505-572):

1. *Zheng J. Recent Construction Technology Innovations and Practices for Large-Span Arch Bridges in China. Engineering. 2024, 41, 110-129.* [*https://doi.org/10.1016/j.eng.2024.05.019*](https://doi.org/10.1016/j.eng.2024.05.019)*.*
2. *Liu K, Wang S. Research on Mechanical Characteristics and Load-bearing Behavior of Nodes of Steel Fiber Concrete Arch Bridges. European Journal of Computational Mechanics. 2023. 32(3), 211-233.* [*https://doi.org/10.13052/ejcm2642-2085.3231*](https://doi.org/10.13052/ejcm2642-2085.3231)*.*
3. *Tan Y, Shi J, Liu P, Tao J, Zhao Y, Santarsiero G. Research on the Mechanical Performance of a Mountainous Long-Span Steel Truss Arch Bridge with High and Low Arch Seats. Buildings. 2023. 13(12).* [*https://doi.org/10.3390/buildings13123037*](https://doi.org/10.3390/buildings13123037)*.*
4. *Zhang Z, Leong E, Gong W, Dai G, Li J. Foundation design of single-span arch bridges in China. Case Studies in Construction Materials. 2023.19.* [*https://doi.org/10.1016/j.cscm.2023.e02239*](https://doi.org/10.1016/j.cscm.2023.e02239)*.*
5. *Qian Y. Comparison and excavation technology of construction schemes for inclined pile arch foundation of long-span arch bridge. National Engineering Construction Industry Construction Technology Exchange Conference. 2021.* [*https://doi:10.26914/c.cnkihy.2021.066161*](https://doi:10.26914/c.cnkihy.2021.066161)*. (In Chinese)*
6. *Li S, Shao Y, Li Y, Huang Z. Deformation Characteristics Analysis of Arch Support Foundation of Super-large Span Arch Bridge Under Complex Rock Foundation Conditions. Highway Engineering. 2025. 1-13. (In Chinese)*
7. *Tian H, Chen W, Cheng P, Yu J. Stablity analysis of rocky slope for arch bridge foundation in gorge area. Rock and Soil Mechanics. 2013, 7. https://doi:10.16285/j.rsm.2013.s1.053. (In Chinese)*
8. *Li S, Liao C, Luo F. Calculation and analysis of arch supported step foundation design in karst areas. Western China Communications Science & Technology. 2020, 3. https://10.13282/j.cnki.wccst.2020.06.026. (In Chinese)*
9. *Yang J, Chen W, Yu H, Tan X. Long-term stability of arch support and bedrock of Daning River Bridge. Chinese Journal of Geotechnical Engineering. 2011, 33, 1178-1184. (In Chinese)*
10. *Zhao Z. STUDY ONLOCAL STRESS ANALYSIS OFCONCRETE FILLED STEELTUBEBUTTERFLY ARCH BRIDGE. Master Degree Thesis. Southwest Jiao tong University. 2011.*
11. *Gorini DN, Callisto L, Whittle AJ. Dominant responses of bridge abutments. Soil Dyn Earthq Eng. 2021, 148. https://10.1016/j.soildyn.2021.106723.*
12. *Gorini DN, Callisto L, Whitttle A. An inertial macroelement for bridge abutments. Géotechnique. 2022, 72, 247-259. https://10.1680/jgeot.19.P.397.*
13. *Al-Homoud AS, Whitman RV. Seismic analysis and design of rigid bridge abutments considering rotation and sliding incorporating non-linear soil behavior. Soil Dyn Earthq Eng. 1999, 18, 247-277. https://10.1016/j.soildyn.2021.106723.*
14. *Balla B, Manandhar S. Analysis of Abutment Foundation using conventional method and FEM model. 2nd International Conference on Earthquake Engineering and Post Disaster Reconstruction Planning (ICEE-PDRP). 2019.*
15. *Xiao T, Pu Q, Zhao Z, Hong Y. Correlation analysis of long-span cable-stayed bridge strain response with environmental and operational variations based on feature selection. Structural Health Monitoring-an International Journal. 2024.* [*https://doi.org/10.1177/14759217241285746*](https://doi.org/10.1177/14759217241285746)*.*
16. *Qiao W, Qi Y, Liu Y. Research on the influence laws of traffic and temperature loads on the strain responses of urban girder bridges. Results in Engineering. 2025, 25.* [*https://doi.org/10.1016/j.rineng.2025.104423*](https://doi.org/10.1016/j.rineng.2025.104423)*.*
17. *Yang M, Cai C, Wei B. A COMBINED CONTROL STRATEGY FOR VIBRATION MITIGATIONS OF A SUSPENSION BRIDGE INDUCED BY VEHICLE BRAKING FORCE. Balt J Road Bridge Eng. 2015. 10, 118-125.* [*https://doi:10.3846/bjrbe.2015.15*](https://doi:10.3846/bjrbe.2015.15)*.*
18. *Wang Y, Li W, Wei N. Dynamic Response Impact of Vehicle Braking on Simply Supported Beam Bridges with Corrugated Steel Webs Based on Vehicle-Bridge Coupled Vibration Analysis. Cmes-Computer Modeling in Engineering & Sciences. 2024. 139(3), 3467-3493.* [*https://doi.org/10.32604/cmes.2024.046454*](https://doi.org/10.32604/cmes.2024.046454)*.*
19. *Zhao Y, Fu Z, Zhu Y, Su T. Reliability Analysis of Degraded Suspenders of Long-Span Suspension Bridge under Traffic Flow Braking. Buildings. 2024. 14(8).* [*https://doi.org/10.3390/buildings14082333*](https://doi.org/10.3390/buildings14082333)*.*
20. *Ge Y, Zheng L, Deng Z, Fang G. Parametric design and numerical analysis of super long span upper-support thrust-bearing concrete arch bridge. Advances in Bridge Engineering. 2025. 6(1).* [*https://doi.org/10.1186/s43251-024-00151-3*](https://doi.org/10.1186/s43251-024-00151-3)*.*
21. *Wang Q, Wan X, Guo H, Qiao H. Dynamic response of long-span arch bridge and riding comfort under wind load. KSCE Journal of Civil Engineering. 2025. 29(2).* [*https://doi.org/10.1016/j.kscej.2024.100021*](https://doi.org/10.1016/j.kscej.2024.100021)*.*
22. *Yang J. Influence zone for end bearing of piles in sand. J Geotech Geoenviron. 2006. 132(9), 1229-1237.* [*https://doi.org/10.1061/(asce)1090-0241(2006)132:9(1229)*](https://doi.org/10.1061/(asce)1090-0241(2006)132:9(1229))*.*
23. *Yu F, Yang J. Base Capacity of Open-Ended Steel Pipe Piles in Sand. J Geotech Geoenviron. 2012. 138(9), 1116-1128.* [*https://doi.org/10.1061/(asce)gt.1943-5606.0000667*](https://doi.org/10.1061/(asce)gt.1943-5606.0000667)*.*
24. *National Railway Administration of the People's Republic of China (NRAPRC). Code for design of building foundation. GB 50007. 2011. Beijing, China. (In Chinese)*
25. *ABAQUS Inc ABAQUS user subroutines reference manual. Dassault Systerms Simulia Corp. 2014. USA.*
26. *National Railway Administration of the People's Republic of China (NRAPRC). Code for Design of Railway Bridge and Culvert. TB 10002-2017. 2017. Beijing, China. (In Chinese)*

5. Specific Comments / Questions

1. How sensitive are the computed stresses to the assumed dilatancy angle and hardening law of the

surrounding mudstone?

**Reply:**

Thanks for your comments. Taking the traffic load conditions S1 as an example, a series of numerical simulations were performed to obtain the calculated stress of mudstone under different dilatancy angle and hardening laws, as detailed below.

Dilatancy angle:

Computed stress of mudstone surrounding arch foundation at different dilatation angles: (a)0°, (b)5°, (c)10°, (d)15°, (e)20°

Hardening laws (Users can specify the relationship between yield stress and absolute plastic strain for compression hardening in ABAQUS software):

Computed stress of mudstone surrounding arch foundation at different hardening laws: (a)yield stress=5 kPa, (b) yield stress=7.5 kPa, (c) yield stress=10 kPa, (d) yield stress=12.5 kPa

It can be observed from the above results that the calculated stress of the mudstone around the arch abutment foundation is not particularly sensitive to the dilatancy angle; in contrast, it is more sensitive to the hardening law of the Drucker-Prager constitutive model to some extent. In the numerical simulations of this study, all parameter settings and boundary conditions are centered around the core of "engineering practice". The authors tend to adopt field actual parameters and environmental conditions, rather than making compromises to achieve a better fitting effect with theoretical values.

2. Does the model include any interface/slip between pile and soil, or is it fully bonded?

**Reply:**

Thanks for your comments. Based on actual engineering conditions, this model allows for slip between the pile and soil interfaces, and related explanations have been added to the revised manuscript based on reviewers' suggestions (page 9, lines 147-149): *General contact is employed across the entire numerical model, with a penalty friction model for tangential contact properties (friction coefficient = 0.55 [24]) and a hard contact model for normal contact.*

The friction coefficient was obtained by surveying technicians after conducting field research. It cannot be directly calculated from the internal friction angle of mudstone because the shear strength between particles does not represent the frictional characteristics of the pile-soil interface. The selection of the coefficient complies with relevant regulations (GB 50007) and comprehensively considers factors such as the roughness of the pile surface, the water content of the soil, and the normal stress. Therefore, it differs from the calculated value (tan30°≈0.577) that usually used in numerical simulations. Similar approaches have been reported in many studies (Skejić et al. 2022; Murali et al. 2022; Mathumidah et al. 2025).

Reference:

National Railway Administration of the People's Republic of China (NRAPRC). Code for design of building foundation. GB 50007. 2011. Beijing, China. (In Chinese)

Skejić A, Gavrić D, Jurišić M, Rahimić Đ. Experimental and numerical analysis of axially loaded bored piles socketed in a conglomerate rock mass. Rock Mechanics and Rock Engineering 2022; 55(10):6339-6365. <https://doi.org/10.1007/s00603-022-02932-6>

Murali AK, Tran KM, Haque A, Bui HH. Experimental and numerical investigation of the load-bearing mechanisms of piles socketed in soft rocks. Rock Mechanics and Rock Engineering 2022; 55(9):5555-5576. <https://doi.org/10.1007/s00603-022-02954-0>

Mathumidah K, Tran KM, Haque A, Bui HH. Effects of Weak-Interface Materials on the Axial Load Capacity of Rock-Socketed Piles: A Numerical Investigation. Rock Mechanics and Rock Engineering 2025:1-24. <https://doi.org/10.1007/s00603-025-04764-6>

3. Since traffic loads vary in time, do the authors expect significant cyclic effects on the plastic zone in

the arch-foot connection area?

**Reply:**

Thanks for your comments. Yes, the authors think that the plastic zone in the arch-foot connection area is subject to significant cyclic effects. However, inferences regarding cyclic effects have not been incorporated into the analysis of Figs. 11 and 16 in the revised manuscript. The authors believe that based on the current nodal loads, it is impossible to conduct accurate analysis or even reasonable inference on the cyclic effects of traffic loads. These aspects have been included in our next research plan, once the main structural form of the bridge is fully finalized. Applying design loads directly to the complete bridge structure seems to be a more appropriate approach to consider the dynamic response of traffic loads, which relies on model tests or numerical simulations.

4. Can the authors share the Abaqus input (*.inp) or CAE file as supplementary data to meet openscience and reproducibility standards?

**Reply:**

Thanks for your comments and suggestions. Following the reviewer's suggestion, the authors have provided the preliminary input file (*.inp) for Case S1 as an attachment within the framework of the competent authority's regulations and ethical guidelines, to meet openscience and reproducibility standards.

5. Were any field measurements (e.g., strain gauges in piles) available for validation?

**Reply:**

Thanks for your comment. The authors believe that field measurements must exist, as field tests are mandatory for main load-bearing components of such extra-long-span bridges. However, as previously mentioned, the entire project has been divided into multiple segments, each conducted independently by several research institutions. Currently, we have not yet obtained the on-site data, and multiple applications for access have been rejected-this is why the numerical model is being verified through theoretical analysis. Furthermore, we must comply with the regulations and ethical guidelines of the competent authorities, which were signed by the authors prior to initiating the research. Furthermore, the authors believe that filet data and some more detailed geological survey data will be obtained upon completion of the entire project, and these data will be made openly available for sharing. Subsequently, authors will conduct more in-depth analyses using these valuable data to advance practitioners' understanding of the mechanical response of arch seat foundations under complex traffic loads.

6. Recommendation

The paper has the potential to be a valuable contribution, but major revision is required to:

• Emphasize novelty and engineering significance,

• Provide clearer details of the constitutive law and load derivation,

• Add discussion that links numerical findings to physical mechanisms and practical design,

• Supply the Abaqus input or CAE file to enhance reproducibility,

• Improve language and figure presentation.
**Reply:**

Thank you very much for your comments and suggestions. The above comments and suggestions have been clearly addressed in the previous responses to the raised issues. The authors greatly appreciate the reviewers for devoting their valuable time to providing highly valuable comments, which has been key to significantly improving the quality of this manuscript and has also offered insights for our future research.

**Reviewer #2:**

1. (a) It is suggested to supplement the name of the finite element analysis software used in Section 3.1.

**Reply:**

Thanks for your comments and suggestions. According to the reviewer's suggestion, the relevant description has been added to the revised manuscript (page 8, line 133): *The finite element analysis in this study was performed using ABAQUS.*

(b)Do both the arch seat foundation and the adjacent slope in Section 3.2 adopt the extended linear Drucker-Prager (DP) model? It is suggested to supplement the explanation.
**Reply:**

Thanks for your comments and suggestions. Not all the adjacent slope adopts the extended linear Drucker-Prager (DP) model. The relevant description has been added to the revised manuscript (page 11, lines 165-167): *To minimize boundary effects, the geometric model is set to a relatively large size, so an elastic constitutive model (0 m-130 m) is used for soil layers at greater distances without affecting the calculation results.*

2. (a) In Fig. 7, the direction of the MAX value coincides with the legend. It is suggested to distinguish them.

**Reply:**

Thanks for your comments and suggestions. Figure 7 in the original manuscript has been revised according to the reviewers' suggestions (page 21, line 307 of the revised manuscript):

**Fig. 11** Maximum principal stress contour plot for the arch seat foundation

(b) In Fig. 8, is the position of the S2 MAX value correct? and supplement the numerical value of S3.

**Reply:**

Thanks for your comments and suggestions. Yes, the position of the S2 MAX value is correct. The original image had an inappropriate viewing angle, which caused visual errors and affected its readability. These issues have been corrected in the revised manuscript (page 23, line 331):

**Fig. 12** Maximum principal stress contour plot for the slanted piles
